# Sustainable Biodegradable Biopolymer-Based Nanoparticles for Healthcare Applications

**DOI:** 10.3390/ijms24043188

**Published:** 2023-02-06

**Authors:** Nika Kučuk, Mateja Primožič, Željko Knez, Maja Leitgeb

**Affiliations:** 1 Faculty of Chemistry and Chemical Engineering, University of Maribor, Smetanova Ulica 17, 2000 Maribor, Slovenia; 2 Faculty of Medicine, University of Maribor, Taborska Ulica 8, 2000 Maribor, Slovenia

**Keywords:** nanoparticles, biopolymers, therapeutic substances, incorporation, biocompatibility, healthcare, biomedical applications

## Abstract

Biopolymeric nanoparticles are gaining importance as nanocarriers for various biomedical applications, enabling long-term and controlled release at the target site. Since they are promising delivery systems for various therapeutic agents and offer advantageous properties such as biodegradability, biocompatibility, non-toxicity, and stability compared to various toxic metal nanoparticles, we decided to provide an overview on this topic. Therefore, the review focuses on the use of biopolymeric nanoparticles of animal, plant, algal, fungal, and bacterial origin as a sustainable material for potential use as drug delivery systems. A particular focus is on the encapsulation of many different therapeutic agents categorized as bioactive compounds, drugs, antibiotics, and other antimicrobial agents, extracts, and essential oils into protein- and polysaccharide-based nanocarriers. These show promising benefits for human health, especially for successful antimicrobial and anticancer activity. The review article, divided into protein-based and polysaccharide-based biopolymeric nanoparticles and further according to the origin of the biopolymer, enables the reader to select the appropriate biopolymeric nanoparticles more easily for the incorporation of the desired component. The latest research results from the last five years in the field of the successful production of biopolymeric nanoparticles loaded with various therapeutic agents for healthcare applications are included in this review.

## 1. Introduction

The field of nanotechnology, which encompasses the synthesis, development, and application of nanomaterials in a size range from 1 to 100 nm, has gained increasing interest in various areas in recent years. Among other things, nanomaterials, especially nanoparticles, play an important role, especially in the field of healthcare applications, where they offer higher efficiency and lower toxicity [1,2,3].

Treatment of various life-threatening diseases, such as chronic diseases (e.g., cancer, heart disease, HIV/AIDS, diabetes) and severe neurological and infectious diseases, is becoming increasingly challenging. Nanomaterials are successfully addressing these challenges by finding new ways for potential treatment, intending to overcome the obstacles of conventional treatment forms [1,2,4]. Various existing formulations for the treatment of certain diseases are poorly soluble, resulting in minimal systemic bioavailability, unstable in vivo, reducing the efficacy of the drug, and causing toxic side effects, even to normal cells. Furthermore, they can cause nephrotoxicity, neurovirulence, and gastrointestinal reactions. Drug resistance may also occur, reducing the impact of further treatment [5].

**Figure 1:**
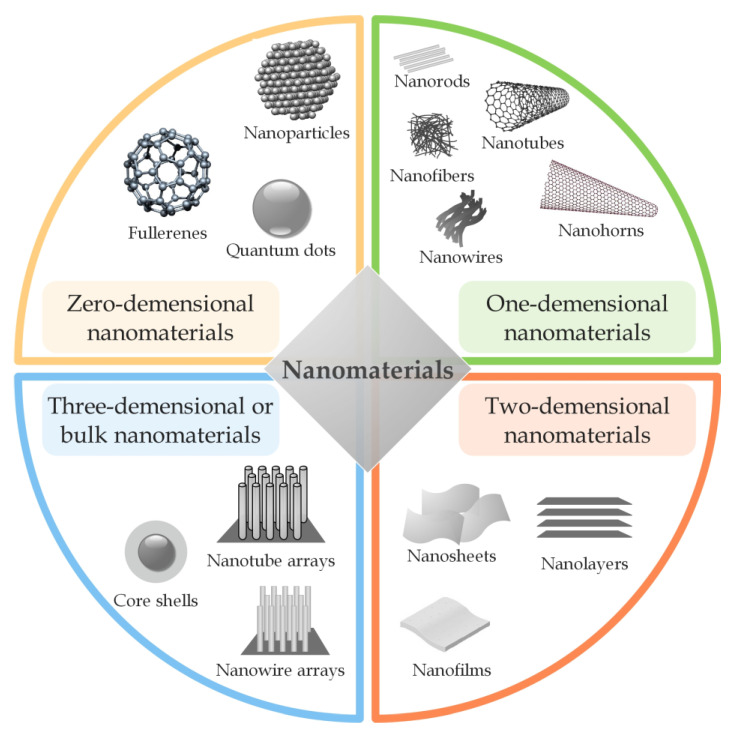
Classification of nanomaterials according to dimensionality (summarized from [2,6,13]).

Nanomaterials are defined as materials with at least one dimension smaller than 100 nm [2,6]. Compared to bulk materials, nanomaterials have different physicochemical properties, such as different shapes, surface area, size, and reactivity, leading to their exceptional performance in many applications. Nanomaterials can be classified according to different criteria [7,8]. One is the classification into four different categories depending on the dimensionality, as shown in Figure 1. Zero-dimensional nanomaterials (0–D) include nanoparticles, fullerenes, and quantum dots. All their three dimensions are in the nanometer range. In one-dimensional nanomaterials (1–D), one dimension is outside the nanoscale, while the other two are inside the nanoscale. This classification includes nanofibers, nanorods, nanotubes, nanowires, and nanohorns. Two-dimensional nanomaterials (2–D), such as nanofilms, nanosheets, and nanolayers, have two dimensions beyond the nanoscale. The most common examples of three-dimensional nanomaterials (3–D) or bulk nanomaterials are core shells, arrays of nanotubes, and nanowires. In this category, the materials are not in the nanoscale in any dimension [2,6,9,10,11,12].

Nanotechnology-based formulations enable better, more effective, and safer use of drugs for the prevention and treatment of various conditions, which is why the production of new nanocarriers for therapeutic agents is becoming increasingly important [1].

A variety of nanoparticles with unique physical and chemical characteristics hold promise for disease diagnosis, biological imaging, and nanomedicine applications [1,14]. They provide the ability to protect the drug from premature degradation, improve intracellular penetration, prolong release, increase absorption and efficacy of the drug at the target site, and therefore represent significant potential in the healthcare sector [4].

It is important to note that the most crucial factor in the production of nanoparticles is to avoid biological and physical barriers such as diffusion, protein adsorption, aggregation, renal or hepatic clearance, and phagocytic sequestration [14].

An important advantage of nanoparticles is that they can be reused after the removal of the incorporated component, which is an important contribution to the circular economy from an economic point of view [15].

**Figure 2:**
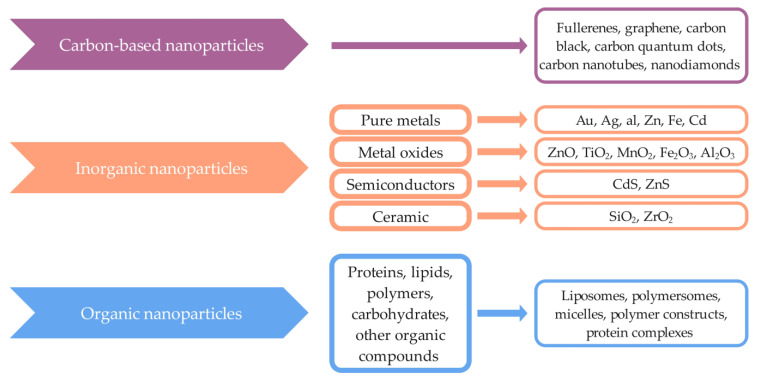
Classification of nanomaterials based on their chemical composition (summarized from [7,16,18]).

Nanoparticles can also be divided according to their composition, meaning according to the material from which they are produced. These include in particular organic, inorganic, and carbon-based nanoparticles (Figure 2) [16]. Carbon-based nanoparticles consist only of carbon atoms and have different morphologies. They are divided into fullerenes, graphene, carbon black, carbon quantum dots, carbon nanotubes, and nanodiamonds [2,7,17,18]. Non-carbon nanoparticles are inorganic nanoparticles and can be produced from pure metals (Au, Ag, Al, Zn, Fe, Cd), metal oxides (ZnO, TiO_2_, MnO_2_, Fe_2_O_3_, Al_2_O_3_), semiconductors (CdS, ZnS), and ceramics (SiO_2_, ZrO_2_) [7,16,19]. Organic nanoparticles include proteins, lipids, polymers, carbohydrates, or other organic compounds and can be classified as synthetic or natural. They are biocompatible, biodegradable, generally non-toxic, and improve pharmacokinetic properties, bioavailability, and drug targeting. Examples are liposomes, polymersomes, micelles, polymer constructs, and protein complexes [2,19,20].

Inorganic nanoparticles, especially metal- and metal oxide-based nanoparticles, are among the most popular. In various applications, such as drug delivery, they are characterized by good stability and half-life of the carrier in the bloodstream, adequate biodistribution, and targeted delivery of the drug to the desired target site [21]. For example, gold nanoparticles are used for numerous diagnostic purposes [22], and silver nanoparticles are also important in biomedicine, primarily because of their antimicrobial potential [23]. Among the metal oxide nanoparticles, ZnO nanoparticles should be highlighted, which are biocompatible, non-toxic, non-expensive, and provide effective antibacterial activity and UV protection [24].

However, they are harmful to the environment, soil, water, and the organisms in them. In addition, some can be very toxic to the human body. Therefore, their use is limited because the body cannot metabolize and excrete them [3]. In contrast, biopolymeric nanoparticles are suitable for various healthcare applications due to their biocompatibility, biodegradability, nontoxicity, and other advantageous properties. The review article covers the latest research results of the last five years on nanoparticles based on natural biopolymers such as proteins and polysaccharides of animal, plant, algal, fungal, and bacterial origin. Special emphasis is paid to the incorporation of various therapeutic agents such as bioactive agents, conventional drugs, antibiotics, and other antimicrobial agents, extracts, and essential oils into biopolymeric nanoparticles. These thus represent promising delivery systems that prevent potential adverse side effects of therapeutic agents, enable targeted delivery to target sites, and thus contribute to enhanced therapeutic efficacy and sustained release.

## 2. Polymeric Nanoparticles

**Figure 3:**
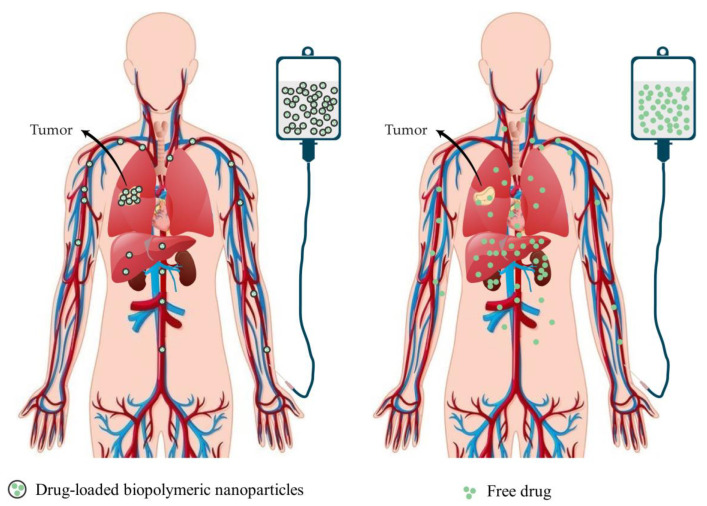
Biodistribution of the drug after intravenous administration when contained in biopolymeric nanoparticles (**left**) compared to the free drug (**right**) (summarized from [30]).

Drug delivery systems must meet specific criteria, such as being biocompatible, biodegradable, stable, non-toxic, non-immunogenic, cost-effective, selective, easy to produce, and delivering and releasing drugs only at specific locations. When using free dosage forms, not only diseased but also healthy cells, tissues, organs, or subcellular organs can be damaged during drug administration and release. Various delivery systems, such as nanoparticles with encapsulated drugs, can successfully circumvent these limitations (Figure 3) [25,26,27]. Drug delivery systems also make it possible to achieve the desired therapeutic effect of drugs and, at the same time, greater efficiency and safety, as well as controlled release of the drug at the target site and prevent aggregation and reduce possible side effects of delivered drugs [25,28,29].

**Figure 4:**
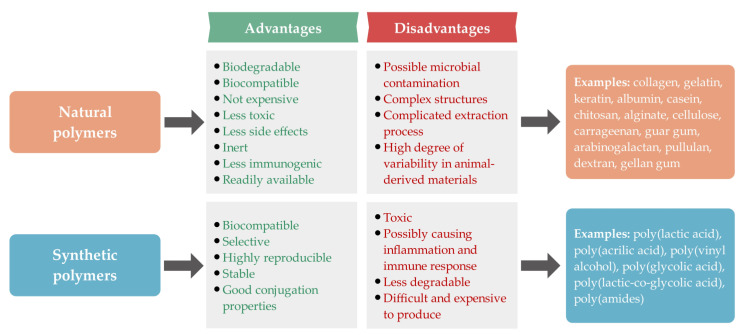
Advantages and disadvantages of natural and synthetic polymers (summarized from [35,36,37]).

Polymer-based nanoparticles are important as alternative carriers for various therapeutic agents for different medicinal purposes mainly due to their biodegradability, biocompatibility, non-immunogenicity, and non-toxicity [29]. Drugs can be incorporated into polymeric nanoparticles by physical or chemical means, i.e., by various adsorption processes on their surface after the nanoparticle formation or by incorporation of drugs into the nanoparticles during the production process. The loading efficacy of therapeutic agents on or into nanoparticles is mainly influenced by nanoparticle size, molecular weight and solubility of drugs, and chemical interactions between drugs and nanoparticles. The release rate of therapeutic agents from polymeric nanoparticles is primarily based on the desorption of surface-bound or adsorbed therapeutic agents, diffusion of therapeutic agents from polymeric nanoparticles, erosion of polymeric nanoparticles, and the combined process of diffusion and erosion [31,32]. Polymeric nanoparticles offer several advantages. They increase the absorption of loaded drugs, provide protection against degradation, prolong the circulation time, and ensure delivery to the target site due to their good solubility, safety and stability, and long-term release [33]. They can be of synthetic or natural origin. However, synthetic polymers can be toxic and immunogenic. Therefore, natural and biodegradable polymers offer better opportunities for nanoparticle production for various biomedical applications [29,34]. Figure 4 shows the advantages and disadvantages schematically and provides examples of synthetic and natural polymers.

### 2.1. Biopolymers Used for the Preparation of Biodegradable Nanoparticles

The most favorable nanoparticles for medical use must meet certain requirements, such as improved bioavailability, protection of therapeutic agents from degradation, controlled drug release, specific targeting, minimal toxicity, stability, and low cost [38], and biopolymeric nanoparticles best meet these requirements.

Biopolymeric nanoparticles comprise biopolymers—natural biological materials that are also generally recognized as safe (GRAS). Biopolymers are macromolecules composed of repeating units of monomers linked together by covalent bonds. Biopolymers such as proteins and polysaccharides are suitable for the preparation of biodegradable polymeric nanoparticles for various biomedical applications, especially for drug delivery, tissue engineering, and wound healing, as the therapeutic effect of the incorporated bioactive substances can be enhanced, and potential side effects can be reduced. This is due to their unique functions and properties, including biodegradability, biocompatibility, photoprotection, and antibacterial and antioxidant properties. In addition, they are naturally renewable, relatively inexpensive, and provide high stability in biological fluids and during storage [3,39,40,41,42,43]. Furthermore, various drugs and therapeutic agents can be successfully incorporated into biopolymeric nanoparticles, contributing to enhanced release at the target site [44]. Both natural polysaccharides and proteins are appropriate carriers for various therapeutic agents in the biomedical field [45]. The use of polysaccharide–protein complexes and conjugates (protein–protein, polysaccharide–polysaccharide, and protein–polysaccharide composite nanoparticles) may be even more efficient due to their additional enhanced properties. Biopolymer composite nanoparticles offer many opportunities to improve biological functions. The preparation of protein–polysaccharide complexes can be manipulated to exhibit improved biocompatibility, biodegradability, stability, targeting, and mechanical properties [46].

**Figure 5:**
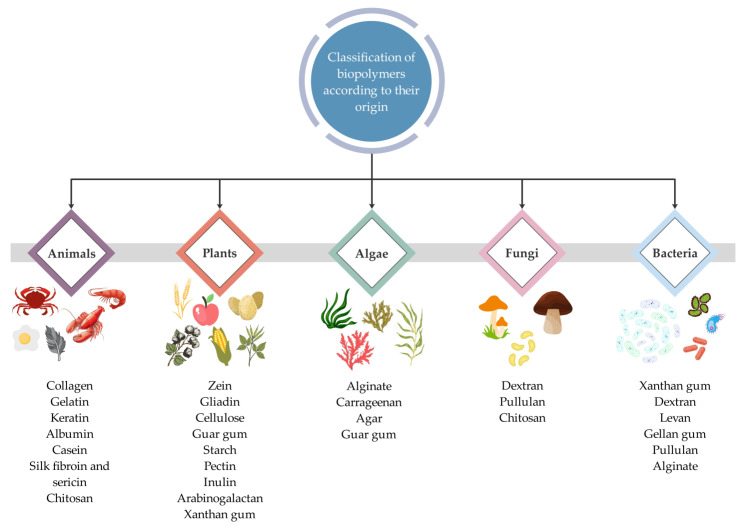
Classification of biopolymers based on their origin (summarized from [47,48]).

Biopolymers can be obtained from various natural sources [37]. Depending on their origin, they can be divided into the animal, plant, algal, fungal, and bacterial biopolymers. Figure 5 shows a schematic representation of the biopolymers classification that can be used for the preparation of biopolymeric nanoparticles, depending on their origin, including the main representatives.

**Figure 6:**
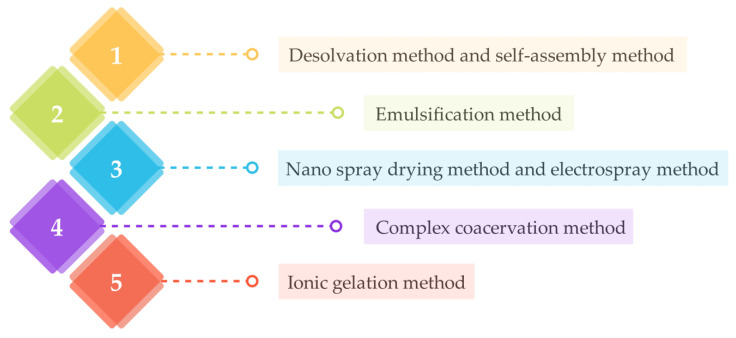
Schematic representation of most commonly used methods for producing biodegradable nanoparticles from biopolymers.

Due to their biodegradability, biocompatibility, nontoxicity, and stability, biopolymeric nanoparticles are promising nanocarrier systems for many drugs and other therapeutic agents. Through different production methods, biopolymeric nanoparticles can be successfully produced. The most commonly used methods are shown schematically in Figure 6. However, the choice of method plays a crucial role in the synthesis of nanoparticles with suitable properties, as it depends on the biopolymer used, the type of therapeutic agent, and the possible route of administration [44,49,50].

The desolvation method is based on using a desolvating (dehydrating) agent, such as ethanol or acetone, which changes the structure of the polymer and reduces its solubility, resulting in the formation of a precipitate of polymeric particles. This method is most commonly used to prepare protein-based nanoparticles [51,52,53]. The self-assembly method depends on the formation of biopolymeric nano-systems by self-assembly, both naturally and artificially, through non-covalent bonds [54,55]. The emulsification method is based on the preparation of nanoparticles by forming an emulsion system by mixing two immiscible liquids, namely the organic and aqueous phases, at very high shear. The organic solvent is then removed by evaporation to form solid nanoparticles [54,56,57]. The nano-spray drying method transforms the material from a liquid into a powder. The solution is sprayed into small droplets through a nozzle into a chamber where drying is carried out by hot gas. The evaporation of the solvent forms dry particles that collect at the bottom of the chamber. The electrospray method is based on manipulating materials on the submicron scale. The polymer solution is sprayed using high voltage through a capillary nozzle that forms aerosolized droplets containing polymer nanoparticles of colloidal size [52,54,58,59]. The complex coacervation method uses pH-dependent electrostatic interactions between biopolymers to form stable nanoparticles and coacervates [52,60]. The ionic gelation method depends on electrostatic interactions between charged polysaccharides in an aqueous medium under certain conditions. It is considered the most commonly used method for the preparation of stable polysaccharide nanoparticles [3,61,62,63].

#### 2.1.1. Protein Nanoparticles

Proteins are biological macromolecules with a high molecular weight, which are inexpensive, abundant, and renewable. Their unique functionalities and properties make them ideal natural materials for the preparation of biodegradable polymeric nanoparticles. They are amphiphilic, which allows good interactions with solvents and various drugs. Protein nanoparticles are produced under mild conditions that do not require the use of toxic chemicals or organic solvents [54,64]. They offer many advantages because they are biocompatible, biodegradable, stable, have non-antigenic properties, are non-immunogenic and non-toxic, and safe.

Moreover, surface modifications of nanoparticles are also possible [52,65]. They are suitable as carriers for various substances, such as natural or chemical therapeutics, growth factors, peptides, vaccines, and nucleic acids. In this way, the potential side effects of the drugs are eliminated, while at the same time, their therapeutic effect at the target site is increased [56]. Therefore, they can be successfully used in various biomedical applications, especially for tissue engineering and the delivery of drugs and other therapeutics [65,66].

Furthermore, various innovative protein-based nanoparticles offer enhanced properties such as biocompatibility, stability, etc. For example, intrinsically disordered proteins (IDPs) are promising nanomaterials for various medical and biotechnological purposes. They can help improve the development of drug delivery systems by enhancing the self-assembly, stimuli-responsiveness, and recognition properties of protein/peptide copolymers [67,68,69,70].

Protein-based nanoparticles can be classified according to the origin of the proteins used for their production. Proteins can be isolated from various animal and plant species. The most commonly used proteins are, for example, collagen, gelatin, and keratin. The composition of proteins depends mainly on the species from which they are obtained. Table 1 lists the most important examples of proteins and their origins suitable for protein-based nanoparticles production.

#### 2.1.2. Polysaccharide Nanoparticles

Polysaccharides are natural biopolymeric materials suitable for biopolymeric nanoparticle fabrication due to their exceptional structural characteristics. They consist of monosaccharide units linked together by glycosidic bonds [79]. Polysaccharides have many advantageous properties, such as biocompatibility, biodegradability, and nontoxicity. In addition, they are relatively inexpensive and readily available [79,80,81]. The presence of hydroxyl groups and other hydrophilic groups, such as carboxyl (e.g., in alginate) and amino groups (e.g., in chitosan), allows easy modification and functionalization of many types of polysaccharides for specific purposes [82,83]. 

Due to various favorable properties, polysaccharide-based nanoparticles are suitable for biomedical applications such as drug delivery and tissue engineering. In addition, they are successfully used as delivery vehicles for different therapeutic agents, including proteins, peptides, and nucleic acids. They can ensure high encapsulation efficiency of various substances and rapid and controlled release at the target site. Furthermore, polysaccharide-based biomaterials have the potential to be used in skin tissue engineering because they can absorb a large amount of water. Due to their biocompatibility and good mechanical properties, they are also increasingly used for bone and cartilage tissue engineering and are also important for the tissue engineering of nerves [80,84].

Polysaccharide nanoparticles can be divided according to the origin of the polysaccharides used for their production [85]. Polysaccharides can be isolated from animal, plant, algal, fungal, and bacterial species. They have different biological characteristics and activities due to different chemical structures and ionic nature [61]. The most well-known natural and biodegradable polysaccharides used in healthcare applications are chitosan, alginate, carrageenan, gellan gum, and dextran [86,87].

Table 2 lists the most important examples of polysaccharides and their origins used for polysaccharide-based nanoparticles production.

## 3. Animal-Based Biopolymeric Nanoparticles Used for Healthcare Applications

Biopolymers of animal origin, such as proteins and polysaccharides, play an essential and versatile role in various biomedical applications, including the production of nanoparticles, especially for drug delivery and tissue engineering.

### 3.1. Protein Nanoparticles of Animal Origin

For the use of biopolymeric nanoparticles for various biomedical applications as carriers of numerous therapeutic substances, proteins of animal origin are most commonly used, of which albumin, casein, collagen, gelatin, keratin, and silk proteins (fibroin and sericin) are the best known.

#### 3.1.1. Albumin

Albumin is the main protein in blood plasma, and due to its many favorable properties, it is a versatile and suitable biomaterial for the preparation of biopolymeric nanoparticles. It is biodegradable, biocompatible, non-toxic, non-immunogenic, available in pure form, highly soluble in water and physiological fluids, and stable in the pH range between 4.0 and 9.0. It also exhibits thermal stability when heated to 60 °C for up to 10 h, as it does not cause denaturation. Albumin can be obtained from a variety of sources, including human, bovine, rat, and chicken, with bovine serum albumin (BSA), human serum albumin (HSA), egg white albumin (ovalbumin), etc. Albumin-based nanoparticles can successfully contribute to increased bioavailability, improved pharmacokinetic properties, and enhanced ability to release incorporated drugs at target sites [51,56,71,128,129]. 

They are successfully used as nanocarriers in the treatment and diagnosis of various diseases, as they can effectively and safely deliver nonspecifically distributed drugs in the body which can cause serious side effects [71,130]. Albumin-based nanoparticles have already been developed for the treatment of cancer, diabetes, hepatitis C, and arthritis [131,132]. Yan et al. [130] prepared albumin nanoparticles loaded with prednisolone and curcumin using a high-pressure homogenization method. Combining two therapeutic substances loaded into albumin-based biopolymeric nanoparticles has shown promise as a delivery system for combined chemotherapy in rheumatoid arthritis. By using biopolymeric nanoparticles, a better anti-inflammatory effect against macrophages was obtained than with a free mixture of drugs. Gawde et al. [133] came to similar conclusions that albumin nanoparticles with incorporated paclitaxel and difluorinated curcumin are suitable for the treatment of gynecologic cancers. Zhao et al. [134] also demonstrated that albumin-based nanoparticles are potential carriers for the co-delivery of paclitaxel and resveratrol for effective synergistic cancer treatment. Moreover, albumin-based nanoparticles produced by the desolvation method with incorporated methylene blue can effectively inhibit the growth of the yeast *Candida albicans* and reduce biofilm formation, as demonstrated in a study by Ambrósio et al. [135]. Using the desolvation method, the most commonly used technique for the preparation of albumin-based nanoparticles, nanoparticles with uniform size, spherical shape, and low aggregation tendency can be produced [135].

#### 3.1.2. Collagen

Collagen is a natural biopolymer and the most abundant protein in the human body, namely the primary material of the extracellular matrix of bones, skin, muscles, cartilage, ligaments, and tendons. It accounts for more than 30% of all body proteins. Due to numerous advantages such as biocompatibility, biodegradability, non-immunogenicity, non-toxicity, tensile strength, low antigenicity, extensibility, and accessibility, it plays an essential role in the synthesis of nanoparticles in various biomedical fields, especially in drug delivery and tissue engineering [136,137,138]. Biodegradable and thermally stable collagen nanoparticles are suitable nanocarriers for many therapeutic and cytotoxic substances, genes, proteins, growth factors, and drugs. In dermatology, for example, they are important for administering antimicrobials and steroids. They have good absorption capacity, large contact surface area, and ensure controlled and long-term release of drugs [136,139,140].

Vijayakumar and Vaseeharan [141] synthesized non-toxic and environmentally friendly collagen-based ZnO nanoparticles with antimicrobial and antibiofilm activity against Gram-positive bacteria *Streptococcus mutans* and Gram-negative bacteria *Proteus vulgaris* and yeast *C. albicans*, and good anticancer activity against human hepatoma cancer cells (HepG2) at a very low concentration (75 μg/mL). Due to their biocompatibility and potential to mimic the extracellular matrix, collagen nanoparticles play an essential role in tissue engineering and regenerative medicine, especially in bone, cartilage, dental, vascular, corneal, and skin tissue engineering, wound healing, spinal cord injury repair, and nerve and oral mucosa regeneration [137,142,143]. In a study by Mondal et al. [144], the chemotherapeutic drug doxorubicin was successfully incorporated into gold-loaded collagen hydroxyapatite nanoparticles. The synthesized nanoparticle system is a promising material for drug delivery and an extracellular matrix for tissue engineering.

#### 3.1.3. Other Animal-Based Protein Nanoparticles

One other important biopolymer for producing nanoparticles of animal origin, which is also promising for nanoparticle fabrication to improve bioavailability, efficacy, and controlled release, is casein, the main protein in milk and a good source of amino acids. Various therapeutic substances, such as the anticancer drug doxorubicin [145], the polyphenolic compounds resveratrol [146], curcumin [147] and quercetin [148], the antibacterial agent mequindox [149], and the phenylpropanoid eugenol [150], have already been successfully incorporated into casein nanoparticles for use in various healthcare applications. Gelatin is another natural, water-soluble protein. Various anticancer drugs, anti-inflammatory drugs, analgesics, antimicrobial agents, and other drugs have already been successfully loaded into gelatin nanoparticles [139,151]. Keratin is a protein rich in amino acids and is fibrous and insoluble. Keratin-based nanoparticles are suitable carriers for various antitumor drugs such as doxorubicin, paclitaxel, and chlorine c6 and 9(R)-9-hydroxystearic acid, and multiple polyphenols such as rutin, quercetin, and curcumin. In addition, they are also crucial for tissue engineering and wound healing [152,153]. Fibroin and sericin are the major proteins of silk proteins. Silk protein nanoparticles are also favorable delivery systems for many drugs, such as paclitaxel, 5-fluoroacil, doxorubicin, gemcitabine, fenofibrate, atorvastatin, methotrexate, inulin, resveratrol, and curcumin [154,155].

### 3.2. Polysaccharide Nanoparticles of Animal Origin

Polysaccharides of animal origin can be successfully used for various healthcare applications, with chitosan being the most important and well-known representative. It is a promising natural biomaterial for the synthesis of nanoformulations for the delivery of different therapeutic agents.

#### Chitosan

Chitosan is a natural biopolymer composed of N-acetyl-D-glucosamine and D-glucosamine units linked by β-1,4-glycosidic bonds. It is a cationic polysaccharide derived from chitin by demineralization and deproteinization. This is found mainly in the exoskeleton of crustaceans and insects, fish scales, and in the cell wall of fungi. Due to its exceptional physicochemical properties such as biodegradability, biocompatibility, non-toxicity, low immunogenicity, mucoadhesiveness, low cost, bio-renewability, and environmental friendliness, chitosan plays an important role in various biomedical and pharmaceutical applications. In addition, they have antitumor, antioxidant, and antimicrobial properties against the growth of Gram-positive and Gram-negative bacteria. Chitosan nanoparticles are stable, increase bioavailability, improve the solubility of poorly soluble drugs, and enhance the targeted delivery of various therapeutic compounds. Chemical modification or integration with functional materials has positive properties to improve in vivo release stability [156,157,158,159,160,161,162,163]. Chitosan-based nanoparticles can be used as nanocarriers for various drug antibiotics, proteins, vaccines, and genes [164,165]. They can be administered by nasal, intravenous, oral, and ocular routes [166].

For example, Ansari et al. [167] synthesized chitosan-based nanoparticles loaded with zinc gluconate using an ionic gelation method. They possess significant antioxidant, anti-inflammatory, and anti-arthritic potential, making them a potential alternative for the treatment of rheumatoid arthritis. Tzeyung et al. [168] prepared rotigotine-loaded chitosan nanoparticles for nose-to-brain delivery using an ionic gelation method. The nanoformulation did not cause toxicity or structural damage to the nasal mucosa, making chitosan-based nanoparticles an effective drug carrier for nose-to-brain delivery as an alternative to conventional administration routes. Yu et al. [169] synthesized dexamethasone–glycol chitosan nanoparticles that exhibited good ocular tolerance and had relatively longer precorneal retention than an aqueous solution. Therefore, these nanoparticles are relevant for ophthalmic drug administration in the treatment of various inflammatory diseases. Chitosan nanoparticles and chitosan/collagen peptide nanoparticles have been shown to be effective biopolymeric stabilizers of Pickering emulsions used for cosmetic applications [170,171]. In addition, chitosan nanoparticles have already been shown to be excellent inhibitors of bacteria, both *Escherichia coli* and *Staphylococcus aureus* [172]. Recent findings include the use of chitosan in the preparation of nanocarriers to enhance the antimetastatic properties of the entrapped drug in vitro and in vivo [173].

Table 3 summarizes examples of biopolymeric nanoparticles of animal origin for various healthcare applications, including their preparation method and the size of the synthesized nanoparticles.

## 4. Plant-Based Biopolymeric Nanoparticles Used for Healthcare Applications

Plants are a rich source of many vital substances, including various polysaccharides and proteins, promising biomaterials for producing biopolymeric nanoparticles for various biomedical applications.

### 4.1. Protein Nanoparticles of Plant Origin

Protein nanoparticles of plant origin are of great importance in the production of biopolymeric nanoparticles because they offer a significant advantage over protein nanoparticles of animal origin. Not only are they much cheaper and widely available, but they are also safer, as diseases from animals can be transmitted to humans through nanoparticles of animal origin. The most important representatives include zein and gliadin.

#### 4.1.1. Gliadin

Gliadin is an important natural protein found in wheat gluten. It can be divided into four classes, namely α-, β-, γ-, and ω-gliadin, according to electrophoretic mobility. The structure of gliadin consists of a terminal region, namely a short N-terminal domain and a C-terminal domain, which is more hydrophobic due to the presence of amino acids, and a central repetitive region rich in glutamine and proline, which confers amphiphilicity to gliadin. Due to its relevant properties, it is suitable for the synthesis of nanoparticles for various therapeutic applications. They are biocompatible, biodegradable, non-toxic, and stable. Due to their hydrophobic nature and low solubility in aqueous solutions, they provide protection and controlled release of the loaded drugs [56,185,186,187]. Gliadin nanoparticles are suitable nanocarriers for various hydrophilic therapeutic agents [188]. Hydrophilic and amphiphilic compounds such as vitamins A and E and amoxicillin have already been successfully incorporated into gliadin nanoparticles [139].

In the study, Alqahtani et al. [189] confirmed that gliadin nanoparticles prepared by a modified antisolvent nanoprecipitation method were compatible with blood regardless of the size of the synthesized nanoparticles. Wu et al. [190] prepared potential delivery agents, resveratrol-incorporated gliadin nanoparticles, by an antisolvent precipitation method and stabilization with gum Arabic and chitosan hydrochloride. The nanocarriers prepared in this way significantly increased the release of resveratrol in water and the simulated digestive tract, thereby increasing its bioavailability. Moreover, they improved the chemical stability and antioxidant activity of the encapsulated polyphenolic compound. Sharif et al. [191] prepared zein nanoparticles with incorporated γ-oryzanol by electrospray method. Due to the encapsulation of γ-oryzanol, the synthesized nanoparticles were rounder and smoother with a homogeneous distribution of γ-oryzanol. The synthesized γ-oryzanol-loaded gliadin nanoparticles had improved stability and decreased the proliferation of HT-29 human colon cancer cells.

#### 4.1.2. Zein

Zein is a major plant-based protein obtained mainly from corn. It is rich in prolamin, poorly soluble in water, and resistant to high temperatures. It is divided into four classes according to different peptide chains, solubility, and molecular size, namely α-, β-, γ-, and δ- zein. Zein is biodegradable, biocompatible, and safe and therefore promising for the synthesis of natural biopolymeric nanoparticles for various healthcare applications. Namely, it offers greater bioavailability, protection, and prolonged, controlled, and more effective release of incorporated substances at the target site. Many therapeutic agents have been successfully loaded into zein-based nanoparticles, e.g., doxorubicin, heparin, 5-fluorouracil, coumarin, quercetin, resveratrol, vitamin D3, and different antimicrobials [185,192,193,194].

Zein-based nanoparticles are applicable nanocarriers for hydrophobic molecules to enhance their bioavailability for various therapeutic purposes. Nunes et al. [195] successfully prepared zein nanoparticles using the nanoprecipitation technique, into which they incorporated resveratrol with an efficiency of 77%. The resveratrol-loaded zein nanoparticles successfully protected resveratrol from enzymatic degradation and showed low toxicity to human colorectal cell lines Caco-2 and HT29-MTX. Using a modified phase separation method, Zhang et al. [193] prepared dodecamer peptide (G23)-functionalized polydopamine (pD)-coated curcumin-loaded zein nanoparticles. The prepared spherical nanoparticles with a diameter of about 120 nm showed excellent cellular uptake by C6 glioma cells and the ability to penetrate 3D tumor spheroids. Zein is also widely used in tissue engineering [192]. Hadavi et al. [196] synthesized zein nanoparticles as a delivery system for a bone morphogenic protein-6 (BMP6) derived peptide using a liquid–liquid phase separation method. They are promising in bone regeneration due to their low toxicity, high encapsulation efficiency, and osteoinductivity.

### 4.2. Polysaccharide Nanoparticles of Plant Origin

Polysaccharides are found in large amounts in various plant species and, due to their non-toxicity compared to various synthetic materials, represent an important natural and biodegradable material for the synthesis of nanoparticles. These nanoparticles are often used as nanocarriers for therapeutic agents for healthcare applications. The best-known polysaccharides of plant origin include arabinogalactan, cellulose, guar gum, inulin, pectin, and starch.

#### 4.2.1. Pectin

Pectin is a structural polysaccharide and consists of the main chain of D-galacturonic acid units linked by α-(1,4) glycosidic bonds and side chains of galactose, arabinose, and rhamnose. It is derived from various plant sources, mainly apples, citrus fruits, and sugar beets, but algae, such as *Spirulina maxima* can also produce it. It is water-soluble, biodegradable, biocompatible, and non-toxic, making it a promising biomaterial for the preparation of nanocarrier systems for various therapeutic agents for biomedical applications [197,198]. Pectin-based nanoparticles are unique systems for targeted drug delivery in the colon because pectin is readily degraded in the colon while remaining chemically intact in the stomach and small intestine [199].

Jacob et al. [200] synthesized polysaccharide nanoparticles based on pectin extracted from citrus fruits using the ionotropic gelation method based on the difference in their degree of esterification, namely high, low, and amidated low methoxylated pectin. Magnesium (Mg^2+^) was used as a divalent crosslinker. All three pectin samples proved to be potential delivery systems for hydrophobic drugs for oral administration. Bostanudin et al. [201] synthesized butylglyceryl-modified pectin nanoparticles by nanoprecipitation. The spherical nanoparticles produced were stable and less than 300 nm in diameter, making them appropriate drug delivery vehicles.

#### 4.2.2. Starch

Starch is a natural biopolymer containing two different polysaccharide structures, amylose, and amylopectin. Amylose is a linear or slightly branched biopolymer and consists of glucose units linked by α-1-4 glycosidic bonds. Amylopectin is a highly branched biopolymer with short chains of glucose units linked by α-(1,4) glycosidic bonds and additional branches linked by α-1-6 glycosidic bonds. Like all other biopolymers, starch is biocompatible, biodegradable, nontoxic, nonimmunogenic, and renewable. Therefore, it represents a significant natural biopolymeric material for the preparation of nanoparticles as successful delivery systems for various drugs and bioactive substances with controlled release at the target site [202,203]. 

Farrag et al. [204] synthesized starch-based nanoparticles of various botanical origins into which the polyphenol quercetin was incorporated using the nanoprecipitation method. The results showed that the synthesized starch nanoparticles had a spherical shape. However, the origin of the starch had an influence on the size of the nanoparticles, the percentage of loading with quercetin, the release kinetics, and the antioxidant activity. Chin et al. [205] successfully synthesized paracetamol-loaded pH-responsive starch–citrate nanoparticles using a nanoprecipitation method. Synthesized nanocarriers are applicable for targeted drug delivery, with relatively high loading capacity and low toxicity. Chen et al. [206] demonstrated that starch-based nanoparticles could be used as nanocarriers for chemotherapeutic agents. In their study, they successfully prepared amphiphilic self-assembled glycyrrhetinic acid–biotin–starch nanoparticles with encapsulated hydrophobic anticancer drug doxorubicin. The nanoformulation showed enhanced cellular uptake and cytotoxic effect on HepG2 cells as a free drug.

#### 4.2.3. Cellulose

Cellulose is a natural structural polysaccharide and the main component of many plant tissues. It is also found in seaweed and various non-pathogenic bacterial species. It is a linear biopolymer composed of D-glucose units linked by β-1,4-glycosidic bonds. Cellulose is the most abundant renewable biopolymer and is biocompatible, biodegradable, non-toxic, and stable under acidic conditions [95,126,207,208]. Cellulose-based nanomaterials (nanocellulose) can be used for a variety of biomedical applications, including wound healing, tissue engineering, drug delivery, bone regeneration, and as antibacterial and antifouling agents [209,210].

Chin et al. [211] demonstrated that curcumin-incorporated cellulose nanoparticles are a promising approach for curcumin delivery to the stomach and upper intestinal tract. In an in vitro study in simulated gastric fluid, higher stability and greater release of the drug were obtained. Cellulose-based nanoparticles are important for achieving target specificity and long-term efficacy in the delivery of therapeutic agents because they can be easily modified to obtain the appropriate chemical, mechanical, and physical properties [209].

#### 4.2.4. Other Plant-Based Polysaccharide Nanoparticles

In addition to pectin and starch, there are other important biopolymers, including arabinogalactan, guar gum, and inulin, which can be used to produce nanoparticles as promising carriers for numerous therapeutics to improve their properties for healthcare applications. Most studies have been conducted primarily on the potential use of biopolymeric nanoparticles to deliver chemopreventive and chemotherapeutic agents [207,212,213]. For example, celecoxib has been successfully incorporated into guar gum nanoparticles to target colon cancer [214], as well as model drugs doxorubicin, 5-fluorouracil, rifampicin, rhodamine B, angiotensin II, antibiotic ciprofloxacin, and polyphenolic compounds, such as curcumin and caffeine. Furthermore, guar gum nanoparticles are suitable for oral as well as buccal, transdermal, and intravenous administration [215]. Inulin nanoparticles have also been developed for efficient delivery of the incorporated drug epirubicin [216]. In addition, inulin-based nanoparticles are potential carriers for unstable molecules in oral delivery systems. They ensure successful encapsulation, protection, and controlled release of quercetin for colon-targeted drug delivery [217]. Moreover, arabinogalactan-based nanoparticles are also suitable as carriers for various therapeutic agents, such as recombinant human thrombin [218].

Table 4 summarizes examples of biopolymeric nanoparticles of plant origin for use in various biomedical applications, including their preparation and the size of the synthesized nanoparticles.

## 5. Algae-Based Biopolymeric Nanoparticles Used for Healthcare Applications

Algae are autotrophs, mixotrophs, and heterotrophs and are found everywhere in freshwater sources and the marine environment. They are divided into microalgae and macroalgae, and both are important for the production of various biopolymers from which nanoparticles can be successfully synthesized [229].

### 5.1. Polysaccharide Nanoparticles of Algal Origin

Many polysaccharides, promising biopolymeric materials for biocompatible, biodegradable, and nontoxic nanoparticles, can also be obtained from algal species. Some important biopolymers of plant origin are also produced by various algae, such as cellulose and starch. Alginate and carrageenan are among the most commonly used biopolymers of algal origin.

#### 5.1.1. Alginate

Alginate is a natural linear and unbranched polysaccharide consisting of a repeating unit of β-D-mannuronic acid (M-blocks) and α-L-guluronic acid (G-blocks) linked by 1,4-glycosidic bonds. The physicochemical properties of alginate are based on the composition, i.e., the M/G ratio and the length and sequence of the building blocks. The high content of M and G blocks contributes significantly to the thickening and gelling properties. Alginate is mainly derived from marine algae but can also be produced by some bacterial species. Due to its important properties, such as biodegradability, biocompatibility, non-toxicity, and non-immunogenicity, alginate is an important natural biomaterial for the preparation of nanoparticles for numerous therapeutic applications. Alginate nanoparticles successfully protect the incorporated therapeutic agents and ensure the controlled release of the therapeutic agent at the target site. The release properties of therapeutic agents are influenced by the presence of monomer units in alginate [127,230,231,232,233,234,235].

Spadari et al. [236] have successfully incorporated miltefosine, a drug with great antifungal potential, into alginate nanoparticles. These have been shown to be alternative nanocarriers for antifungal drugs to treat invasive fungal infections without having a cytotoxic effect on healthy cells, as may be the case when the free drug is administered. The bacteriocins of *Lacticaseibacillus paracasei* CNCM I-5369 show good activity against Gram-negative bacteria, including *E. coli*, which contains the mcr-1 gene that makes it resistant to the antibiotic colistin. In a study, Belguesmia et al. [237] successfully loaded alginate nanoparticles with bacteriocins, providing a successful therapeutic alternative in the fight against infections caused by Gram-negative bacteria. The results showed that antibacterial activity against *E. coli* increased significantly. Moreover, the alginate nanoparticles loaded with bacteriocins were not cytotoxic to human HT29 and animal IPEC-1 cell lines.

#### 5.1.2. Carrageenan

Carrageenan is a linear sulfated polysaccharide obtained mainly from various species of seaweed. It is a hydrophilic biopolymer with a high molecular weight. Its polymer structure consists of 3-linked-β-D-galactopyranose and 4-linked-α-D-galactopyranose or 4-linked 3,6-anhydro-α-D-galactopyranose units. According to the structural features of the repeating disaccharide units, it can be classified into seven types, namely λ-, κ-, ι-, υ-, μ-, θ-, and ξ-carrageenan. Due to biodegradability, biocompatibility, bioavailability, non-toxicity, and thermostability, carrageenan is the ideal basis for the production of important nanoparticles based on natural polysaccharides in biomedical applications. It significantly contributes to improving the properties of incorporated therapeutic substances, increases bioavailability and durability, and prolongs the release of bioactive ingredients at the target site. In addition, carrageenan also has antioxidant, anticarcinogenic, anticoagulant, and immunomodulatory properties [61,238,239,240,241].

Fani et al. [242] used an electrospray method to synthesize carrageenan nanoparticles loaded with the sensitive bioactive compound D-limonene (R-(+)-limonene) and achieved an encapsulation efficiency of about 97%. Carrageenan-based nanoparticles were found to be effective nanocarrier systems for lipophilic compounds such as D-limonene, as both photostability and thermostability were significantly improved by encapsulation. Vijayakumar et al. [243] synthesized carrageenan-coated ZnO nanoparticles with successful antibacterial activity against MRSA. Moreover, the prepared nanoparticles had proven anti-inflammatory activity, good compatibility with human red blood cells, and were environmentally safe.

Table 5 summarizes examples of algae-based polysaccharide nanoparticles for various healthcare applications, including preparation methods and the size of produced nanoparticles.

## 6. Fungal-Based Biopolymeric Nanoparticles Used for Healthcare Applications

Fungi are an essential source of various natural compounds, including polysaccharides. Biodegradable and biocompatible nanoparticles, as carriers of drugs and other important therapeutic agents, can be synthesized from successfully extracted biopolymers from fungi by various methods.

### Polysaccharide Nanoparticles of Fungal Origin

Pullulan is one of the most important and commonly used fungal polysaccharides for the preparation of biopolymeric nanoparticles. In addition to pullulan, the polysaccharide dextran, mainly of bacterial origin, and the protein chitosan, primarily derived from animals, can also be isolated from fungi.

#### Pullulan

Pullulan is a natural biopolymer obtained mainly by a fermentation process from the yeast-like fungus *A. pullulans*. The structure of pullulan contains maltotriose units linked by α-1,6 glycosidic bonds, which contributes significantly to the structural flexibility of pullulan. It is a nonionic linear exopolysaccharide easily soluble in water but insoluble in most organic solvents. Compared to other polysaccharides, the aqueous pullulan solution is stable and has a relatively low viscosity. Due to its advantageous properties, such as biodegradability, biocompatibility, non-immunogenicity, non-toxicity, non-mutagenicity, and non-carcinogenicity, pullulan is an important biomaterial for the preparation of nanoparticles based on natural polysaccharides, applicable for various biomedical use [61,111,249,250,251]. Pullulan-based nanoparticles are nanocarriers for various therapeutic substances, for example, antimicrobial agents and essential oils with inhibitory activity against pathogenic Gram-negative and Gram-positive bacteria and fungi, including multidrug-resistant and biofilm-forming bacteria [252].

Tao et al. [253] synthesized potential biopolymer-based nanocarriers to reduce the dose of chemotherapeutic drugs while increasing their delivery efficiency. They successfully incorporated the drug mitoxantrone into cholesterol-substituted pullulan nanoparticles, which showed successful growth inhibition of bladder cancer cells in vitro. Yuan et al. [254] synthesized cholesteryl-modified aminated pullulan nanoparticles loaded with cholesterol succinate, an acidic cholesterol ester. Sustained release of incorporated substance and cytotoxicity on Lewis lung cancer cells were obtained. In a study, Laha and Maiti [255] found that stearyl pullulan-based nanocarriers with the incorporated drug glipizide could prolong the release of the loaded drug under simulated gastrointestinal conditions. Thus, they proved to be favorable nanocarriers for poorly soluble drugs that can achieve controlled release and successfully improve the therapeutic efficiency of the incorporated drugs. Li et al. [256] demonstrated that pullulan–doxorubicin nanoparticles loaded with 1,1,2-trichlorotrifluoroethane are a new potential delivery system with a synergistic effect that significantly improves ablation and therapeutic efficacy.

Table 6 summarizes examples of polysaccharide nanoparticles of fungal origin for use in various biomedical applications, including synthesis methods and the size of nanoparticles produced.

## 7. Bacterial-Based Biopolymeric Nanoparticles Used for Healthcare Applications

Several bacterial species can produce various bioactive compounds, including polysaccharides, from which biodegradable, biocompatible, and nontoxic nanocarriers for potential use in biomedical applications can be prepared.

### 7.1. Polysaccharide Nanoparticles of Bacterial Origin

Polysaccharides that are successfully used to produce biopolymeric nanoparticles of bacterial origin include dextran, xanthan gum, levan, and gellan gum, of which dextran is the best known. In addition, pullulan can also be of bacterial origin. Polysaccharide-based nanoparticles of bacterial origin can successfully enhance bioavailability and consequently reduce the side effect of the incorporated drug or other therapeutic substance.

#### 7.1.1. Dextran

Dextran is a natural, branched exopolysaccharide with a hydrophilic nature. It is produced mainly by some Gram-positive bacteria such as *Leuconostoc* and *Streptococcus*. The main dextran chain consists of glucose monomers linked by α-1,6 glycosidic bonds and short-side branches linked by α-1,3 glycosidic bonds. However, the properties of dextran, such as branching and molecular weight, may vary depending on the bacterial strain and production conditions. Due to its advantageous properties, such as biodegradability, biocompatibility, non-toxicity, and non-immunogenicity, it is considered as a suitable biomaterial for the preparation of polysaccharide nanoparticles as delivery systems for various therapeutic agents, including proteins and nucleic acids. Moreover, dextran nanoparticles successfully reduce the potential negative side effects of incorporated substances by delivering them to the target sites in a controlled manner [262,263,264].

In order to minimize the local and systemic side effects of local retinoblastoma chemotherapy, Delrish et al. [265] synthesized thiolated and methylated chitosan–carboxymethyl dextran nanoparticles by the ionic gelation method. They studied their biodistribution after intravitreal injection into the eyes of rats with retinoblastoma. The diameter of the prepared biocompatible polymer nanoparticles was 34 ± 3.78 nm and 42 ± 4.23 nm, respectively. These showed increased ocular bioavailability in retinoblastoma-induced rat eyes. In a study, Han et al. [266] found that paclitaxel-loaded dextran nanoparticles decorated with the RVG29 peptide can cross the blood–brain barrier and reach intracranial tumors. Therefore, they are suitable drug delivery systems for the treatment of C6 glioma. Jamwal et al. [267] synthesized a dextran-based insulin delivery system. An in vitro insulin release study was performed in an artificial gastric and artificial intestinal fluid and showed controlled release of insulin from synthesized glucose oxidase immobilized-acryloyl crosslinked dextran dialdehyde nanoparticles.

#### 7.1.2. Other Bacterial-Based Polysaccharide Nanoparticles

Other bacterial-derived polysaccharides, such as xanthan gum, levan, and gellan gum, are also nontoxic, biodegradable, and biocompatible polymers for the synthesis of biopolymeric nanoparticles promising for biomedical applications as delivery systems. Therefore, various therapeutic substances have already been successfully incorporated into bacterial-based polysaccharide nanoparticles. For example, gellan gum and levan nanoparticles have been successfully loaded with resveratrol for oral administration [268,269], and the hydrophilic antihypertensive drug Atenol was incorporated into gellan gum nanoparticles [270]. Moreover, a chemotherapy drug, doxorubicin, was also successfully incorporated into xanthan gum nanoparticles [271].

Table 7 summarizes examples of polysaccharide nanoparticles of bacterial origin for use in various healthcare applications, including preparation methods and size of the synthesized nanoparticles.

## 8. Incorporation of Different Therapeutic Substances into Biopolymeric Nanoparticles for Healthcare Applications

Biopolymeric nanoparticles are applicable for clinical use as delivery systems for various drugs, genes, and other therapeutic agents and tissue engineering. This is due to their favorable properties, especially biodegradability, biocompatibility, non-toxicity, and low induction of immune responses [278,279]. Since they allow targeted delivery of incorporated drugs to target sites, preventing toxic effects on healthy cells, they can be loaded with a high concentration of drugs, allowing enhanced therapeutic effects due to sustained release. The loaded drug is successfully protected from metabolic processes and thus retains its bioactivity [3,84]. 

Free drugs require high dosages to achieve a pharmacological effect and are potentially toxic. In contrast, biodegradable biopolymer nanocarriers represent unique and effective systems for various healthcare applications due to their favorable properties and enhanced drug delivery in the human body. Moreover, they successfully reduce the potential risks and drawbacks of conventional therapies. There are several routes of administration by which therapeutic agents can be introduced into the human body using biopolymeric nanoparticles, namely the enteral (oral, ocular, nasal) or parenteral (subcutaneous, intravenous, intramuscular) route. However, the appropriate route of administration is influenced by the disease state, the desired therapeutic effect, and the bioavailability of the drug [278,280]. 

### 8.1. Incorporation and Delivery of Bioactive Compounds

Biologically active compounds, such as polyphenolic compounds, have antioxidant, anti-inflammatory, antimicrobial, antitumor, antiallergic, and other beneficial biological activities. However, they are susceptible to environmental conditions such as light, heat, and oxygen and are, therefore, chemically unstable. Furthermore, they are poorly soluble in water and are rapidly metabolized [49]. These limitations can be successfully overcome by incorporating bioactive compounds into nanocarriers, such as biopolymeric nanoparticles [195]. It has already been confirmed that encapsulation of phenolic compounds in nanoparticles significantly improves their stability, water solubility, and bioavailability, as well as their biological activity. Therefore, biopolymeric nanoparticles are suitable carriers for these compounds, as they can also reduce the required dose of bioactive compounds, further reducing potential side effects [49].

Many bioactive compounds also have good antioxidant properties. However, their potential use in the treatment of diseases caused by oxidative stress, including neurodegenerative diseases, is a significant concern because brain targeting is still a major challenge. Nanoformulations with incorporated antioxidants are promising delivery systems because they protect antioxidant molecules from degradation while allowing improved bioavailability and targeted delivery to specific organs, cells, or tissues [31].

The bioactive ingredient curcumin has many beneficial properties for human health. Therefore, many studies have already been conducted on its encapsulation in various biopolymeric nanoparticles to provide protection from rapid degradation and enhance its therapeutic effects. Das et al. [281] successfully incorporated curcumin into albumin-based nanoparticles, which showed enhanced cellular uptake and cytotoxicity in human lung carcinoma cells (A549) compared with the free compound. Hassani et al. [282] successfully encapsulated curcumin into sodium alginate–gum Arabic nanoparticles for enhanced antioxidant and drug-release properties of curcumin. The curcumin alginate–gum Arabic nanoparticles were prepared by an ionotropic gelation technique with slight modifications using calcium chloride as a crosslinker. Curcumin-loaded nanoparticles have significant therapeutic potential in preventing and treating solid malignancies. Compared with free curcumin, they showed enhanced anticancer activity against HepG2, HT29, A549, and MCF-7 cancer cells, as demonstrated by a cytotoxicity study using the MTT assay. Nogueira et al. [283] have also successfully incorporated curcumin into biopolymeric nanoparticles based on the polysaccharide carrageenan. High hydrophobicity may hinder the in vivo bioavailability of curcumin, reducing its therapeutic effect. Consequently, its incorporation into biodegradable nanoparticles is crucial. Curcumin-loaded carrageenan nanoparticles are a potentially effective and safe system for the treatment of bone-related diseases. Starch nanoparticles can also be used as nanocarriers for curcumin. Acevedo-Guevara et al. [222] used natural and acetylated starch from green bananas to prepare nanoparticles. Curcumin was successfully incorporated into both nanoformulations with an encapsulation efficiency greater than 80%. However, the acetylated starch nanoparticles were more successful because the acetylation of starch caused stronger hydrogen bond interaction between curcumin and the starch matrix, making the encapsulation more effective. In addition, improved controlled release of the polyphenolic substance from the acetylated starch nanoparticles was achieved. By incorporating curcumin into ovalbumin/κ-carrageenan nanoparticles, Xie et al. [284] demonstrated high antioxidant activity and stability in simulated gastric fluid and enhanced thermal and light stability of curcumin. Fan et al. [285] synthesized nanoparticles based on BSA and dextran for the preparation of curcumin delivery systems. The results of the study showed that the prepared nanoparticles loaded with curcumin were stable. Further, the antioxidant activity of curcumin in Caco-2 cells compared to the free bioactive compound was significantly improved. Liu et al. [286] synthesized zein nanoparticles with incorporated curcumin and stabilized dual-coating shell structure in combination with sodium caseinate (SC) and sodium alginate (SA). Zein nanoparticles stabilized with SC-SA significantly improved the photochemical stability and antioxidant activity of curcumin and enabled controlled release under simulated gastrointestinal conditions. The nanocarriers prepared in this way, therefore, have the potential to successfully deliver chemically unstable hydrophobic bioactive compounds. They can improve the water solubility, photochemical stability, and antioxidant activity of bioactive compounds such as curcumin. The same conclusions were reached by Yao et al. [287], who used zein nanoparticles loaded with curcumin and an alginate/gelatin biopolymer coating in their study. Biopolymeric materials can form stable coatings on the surface of zein and other nanoparticles made of natural polymers, which contributes to improved pH stability of nanoparticles and inhibition of particle aggregation during long-term storage [192,287]. Sorasitthiyanukarn et al. [288] successfully prepared chitosan/alginate nanoparticles loaded with curcumin diglutaric acid, a prodrug of curcumin with enhanced solubility and antinociceptive activity. These were more stable than free curcumin diglutaric acid under UV radiation and in simulated gastrointestinal environments. Additionally, they showed enhanced anticancer activity against Caco-2, HepG2, and human breast cancer cells (MDA-MB-231), as well as higher in vitro cell uptake in Caco-2 cells. Therefore, the authors claim that chitosan/alginate nanoparticles are a potential system for the oral delivery of curcumin diglutaric acid for cancer treatment. In a more recent study [289], the same authors synthesized chitosan/alginate nanoparticles loaded with curcumin diethyl disuccinate, an ester prodrug of curcumin. Compared with the free therapeutic substance, these showed improved stability, bioavailability, bioaccessibility, cellular uptake, and cytotoxicity against HepG2. In addition, chitosan-κ carrageenan nanoparticles with incorporated α-mangostin, a xanthone derivative isolated from the pericarp extract of mangosteen (*Garcinia mangostana* L.), are also promising delivery systems exhibit anticancer and excellent physicochemical properties [290]. They showed increased solubility, prolonged release, and cytotoxicity against MCF-7 cancer cells.

Rathore et al. [291] incorporated silymarin, a flavonoid, into collagen-based nanoparticles. Silymarin is a bioactive compound with hepatoprotective and neuroprotective properties isolated from milk thistle. The silymarin-loaded nanoformulation showed improved therapeutic efficacy of the flavonoid against acute ischemia/reperfusion injury. Moreover, a lower drug dose can achieve an appropriate neuroprotective effect by using nanoparticles. In addition, Roy and Rhim [292] incorporated a natural antioxidant flavonoid quercetin into chitosan-based nanoparticles, which exhibited good antioxidant and antimicrobial properties. Quercetin was also successfully loaded into zein nanoparticles stabilized with soluble soybean polysaccharide. This nanoformulation significantly improved the encapsulation efficiency and photochemical stability of quercetin [293]. Cinnamaldehyde is another natural compound belonging to flavonoids. Subhaswaraj et al. [182] prepared chitosan nanoparticles loaded with cinnamaldehyde, which showed significantly higher anti-quorum sensing activity and antibiofilm activity against *Pseudomonas aeruginosa* compared to free cinnamaldehyde. Moreover, slow and sustained release of cinnamaldehyde was achieved in an in vitro release study. Liang et al. [294] incorporated the polyphenolic compound tannic acid into synthesized zein/pectin nanoparticles with an average diameter of 166 nm and achieved an encapsulation efficiency of 89%. The results showed good antioxidant activity of the incorporated tannic acid, gradually released from the nanoparticles under simulated gastrointestinal conditions, especially in the small intestine.

Anacardic acid is a hydroxybenzoic acid with exceptional antimicrobial properties. Araujo et al. [295] successfully loaded anacardic acid into zein nanoparticles using a nanoprecipitation method. This method is considered an economical, environmentally friendly, low-energy, and reproducible technique. Nanoencapsulation of anacardic acid even increased antimicrobial activity against *S. aureus*, *P. aeruginosa*, and *Candida auris*. In another study, zein nanoparticles loaded with anacardic acid showed high bactericidal activity against *S. mutans* and potential antiplaque activity [296]. Bernal-Mercado et al. [297] incorporated carvacrol, a monoterpenoid phenol extracted from oregano and thyme essential oils, into chitosan nanoparticles, enhancing their antibacterial and antibiofilm properties against *P. aeruginosa*. Furthermore, Jain et al. [298] successfully loaded beta-carotene into zein nanoparticles prepared by a modified phase separation technique. They demonstrated enhanced cellular uptake, cytotoxicity, and improved oral biopharmaceutical performance of beta-carotene.

### 8.2. Incorporation and Delivery of Conventional Drugs

Selective therapeutics targeting diseased areas is a significant challenge in the treatment of many diseases, especially various forms of cancer [299]. Different anticancer drugs are poorly soluble in water and may have multiple toxic side effects, which can be successfully eliminated by incorporation into different nanocarriers [219]. Therefore, chemopreventive and chemotherapeutic agents can be successfully encapsulated into various biopolymeric nanoparticles. These offer improved stability and solubility of the drug, as well as a longer half-life in the blood due to protection from modifications caused by metabolic enzymes. In addition, drug encapsulation successfully reduces drug resistance and enables controlled drug release at the target site [31]. Nanoparticles have the ability to deliver incorporated drugs to the target organ, cells, or tissues, preventing the potentially toxic effects of drugs on healthy cells, as is the case with conventional forms of drug delivery. As a result, the amount of drug administered can also be reduced to achieve the desired therapeutic effect [300]. They are also potential gene nanocarriers due to their nanoscale size, high surface-to-volume ratio, and stability [31].

Yu et al. [219] successfully incorporated the anticancer drug maytansine with an encapsulation efficiency of 82.97% into zein nanoparticles prepared by phase separation to target the tumor and reduce the toxic side effects of the drug. Maytansinin-loaded zein nanoparticles have been shown to be non-toxic and promising nanocarriers for the treatment of non-small cell lung cancer, demonstrating enhanced antitumor efficacy in A549 cells compared to the free drug. Another anticancer drug, paclitaxel, was successfully incorporated into potato starch nanoparticles by Putro et al. [225]. The cytotoxicity assay results showed good biocompatibility of the nanoparticles with 7F2 mouse osteoblast cells. Abid et al. [299] synthesized enzyme-responsive dextran-based oligoester crosslinked nanoparticles containing the anticancer drug 5-fluorouracil as a potential oral delivery system to combat colon cancer. In a study by Ding et al. [224], 5-fluorouracil was also successfully incorporated into biopolymeric particles. The synthesized retrograded starch nanoparticles showed high absorption capacity and long in vitro release of the loaded drug, making them promising nanocarriers for colon-specific controlled release of 5-fluorouracil.

Pirfenidone is a drug with anti-inflammatory and antifibrotic activity used to treat idiopathic pulmonary fibrosis. Abnoos et al. [301] incorporated pirfenidone into chitosan/alginate nanoparticles with an encapsulation efficiency of 94%. An in vitro drug release study showed sustained release of the encapsulated drug, and skin penetration of the nanoparticle-loaded pirfenidone was compared to the free drug. Kianersi et al. [245] prepared alginate nanoparticles coated with chitosan and gelatin and loaded with the anti-inflammatory drug betamethasone sodium phosphate.

Thomas et al. [302] prepared starch-modified alginate nanoparticles using the ionotropic gelation method, into which they incorporated the drug theophylline and the protein BSA as model drugs. In vitro cytotoxicity studies showed that the nanoparticles were completely nontoxic and exhibited excellent biocompatibility. Nanoparticles prepared in this way successfully protected the incorporated drug in the acidic environment of the gastric fluid, and complete release of the drug in the intestinal fluid was achieved. Constantin et al. [303] prepared spherical nanoparticles based on a pullulan derivative incorporated with the model drug sodium diclofenac. In vitro studies revealed that the polymeric nanoparticles did not exhibit cytotoxicity at a pharmacologically relevant concentration of diclofenac and retained typical cell morphology. 

Ursodeoxycholic acid is an oral drug for the treatment of primary biliary cirrhosis. Yu et al. [257] successfully demonstrated the neuroprotective effect of ursodeoxycholic acid-loaded pullulan acetate nanoparticles stabilized by poly(vinyl alcohol). Chinnaiyan et al. [221] prepared metformin-loaded pectin-based nanoparticles with an encapsulation efficiency of 68% by an ionic gelation method. The results showed reasonable stability in the presence of excess BSA and slow and sustained release of the drug at pH 6.8. In vivo stability of the nanoparticles and the possibility of prolonging the retention time of the drug in the bloodstream were demonstrated. Therefore, the pectin nanoparticles developed in this way are appropriate for enhancing the bioavailability of metformin for the successful treatment of type 2 diabetes.

### 8.3. Incorporation and Delivery of Antibiotics and Antimicrobial Agents

Due to increasing resistance to antibiotics and other antimicrobial agents, the search for new formulations for their administration has become increasingly important. Various metal nanoparticles are considered effective inhibitors of bacterial growth but are also highly toxic to human cells [304,305]. On the other hand, biocompatible and non-toxic biopolymeric nanoparticles also exhibit antimicrobial activity against various bacterial species [3]. Different nanoformulations, including polymeric nanoparticles in addition to liposomes, micelles, and solid lipid nanoparticles, can be considered suitable nanocarriers for antibiotics and other antimicrobial agents. Incorporating antibiotics into suitable nanoparticles can overcome antibiotic resistance, which increases the efficacy against pathogenic bacteria and contributes to the controlled release of the drug at the site of infection. In addition, encapsulation of antibiotics also has a positive effect on overcoming certain limitations, including poor solubility and stability of the drug and low permeation through biological barriers [306,307,308]. Thus, they can protect antibiotics from pH and/or enzymatic degradation [309]. 

Biopolymeric nanoparticles have been shown to be effective antibiotic carriers for combating antibiotic resistance. In vitro studies have demonstrated superior antibacterial activity against clinical isolates of multidrug-resistant *E. coli*, *Klebsiella pneumoniae*, and methicillin-resistant *S. aureus* (MRSA) compared to free antibiotics [306]. Furthermore, several antibiotics, such as colistin, can be toxic and provide low tissue penetration, so they are not commonly used. However, Scutera et al. [306] successfully incorporated the antibiotic colistin into HSA-based nanoparticles coated with chitosan. Compared to the free antibiotic, high antibacterial activity against the growth and inhibition of biofilm formation of multidrug-resistant Gram-negative bacterial species responsible for hospital-acquired severe infections (*Acinetobacter baumannii* and *K. pneumoniae*) was demonstrated. The antibacterial activity of the antibiotic imipenem and ciprofloxacin separately and in combination incorporated into albumin nanoparticles was increased, particularly against methicillin-resistant *S. aureus* and *S. mutans* [176]. Alginate/chitosan nanoparticles with incorporated antimicrobial drug tobramycin could be effectively used in the treatment of chronic infection with *P. aeruginosa* [310].

Furthermore, Liu et al. [311] prepared chitosan–sodium alginate nanoparticles into which they incorporated ε-polylysine, a homo-poly amino acid. In antimicrobial studies against the growth of *E. coli*, *S. aureus*, *B. subtilis*, and *Micrococcus luteus*, they showed three times higher bacteriostatic activity than free ε-polylysine. In vitro studies also showed prolonged release of the component from the nanoparticles. In patients with recurrent pulmonary infections, intrapulmonary administration of antibiotics may be beneficial compared with intravenous injection. Namely, inhalation can achieve a faster onset of action and a higher concentration of therapeutic agents in the lungs. Therefore, Falciani et al. [312] prepared a nanoformulation by binding the SET-M33 peptide, a not natural antimicrobial peptide, to single-chain dextran nanoparticles. Nanoparticles have been shown to be effective against pneumonia-causing *P. aeruginosa* in a mouse model by intrapulmonary administration. In addition, the retention time of the SET-M33 peptide in the lungs was effectively improved by incorporation into dextran-based nanoparticles. Moreover, Yu et al. [313] synthesized conjugates of chitosan nanoparticles and the antimicrobial peptide microcin J25. These have significantly enhanced antimicrobial activity against Gram-negative and Gram-positive bacteria while causing no toxicity in HEK293 T and Caco-2 cell lines.

### 8.4. Incorporation and Delivery of Extracts

It is known that natural extracts contain various biologically active substances that have a positive effect on health. Since they can be degraded in the gastrointestinal tract, their bioavailability is often poor. For the protection of sensitive compounds, the encapsulation of the extracts in different nanocarriers is crucial. A sustainable and suitable option are natural, non-toxic, biodegradable, and biocompatible protein- or polysaccharide-based nanoparticles to enhance their bioavailability and overcome biological barriers [246].

For this purpose, Costa et al. [246] successfully incorporated bioactive grape pomace extract into alginate and chitosan nanoparticles and investigated their potential as suitable nanocarriers for oral delivery. Alginate and chitosan nanoparticles protected the extract from the gastrointestinal environment, increasing the bioavailability of polyphenolic compounds in the extract and thus enhancing antioxidant and antimicrobial activity. In addition, the encapsulation of the extract also reduced its permeability through the intestinal barrier, allowing the extract to remain in the intestine longer, thus improving the prebiotic potential of the extract. Rahimivand et al. [314] successfully synthesized alginate nanocarriers with an incorporated ethanolic extract of *Artemisia ciniformis*, which increased cytotoxicity and induced apoptosis against AGS gastric cancer cells compared with the free extract. Encapsulation successfully protected the extracts from environmental stress. Wang et al. [223] synthesized starch nanoparticles obtained from ginkgo and corn, respectively, loaded with *Ginkgo biloba* extract using the nanoprecipitation method. Starch nanoparticles proved to be promising delivery systems for *Ginko biloba* extract or other bioactive compounds. The extract-loaded starch nanoparticles showed sustained release compared to the free extract in artificial gastric and intestinal juices. Additionally, Nallasamy et al. [226] have prepared starch-based nanoparticles into which they have successfully incorporated Triphala Churna extract, a polyherbal formulation rich in polyphenols and vitamin C that has rejuvenating and anti-aging effects. Encapsulation in biopolymer nanoparticles increased the stability, solubility, and efficacy of the Ayurvedic drug and achieved sustained release. In addition, antioxidant and anticholinesterase activities were maintained. A strong antimicrobial activity against *Salmonella typhi*, *Shigella dysenteriae*, and *S. aureus*, as well as anti-biofilm activity against ATCC MRSA 33591 and clinical strain N7 were demonstrated. Soltanzadeh et al. [183] synthesized spherical, physically stable chitosan nanoparticles with a diameter of 174–898 nm, into which pomegranate peel extract was successfully encapsulated. The study indicates promising nano-delivery systems for extracts and the resulting protection and controlled delivery of natural, sensitive substances with antioxidant and antimicrobial properties. Furthermore, Beconcini et al. [315] incorporated cherry extract into chitosan nanoparticles, successfully protecting the antioxidants contained in the extract from degradation in the gastrointestinal tract.

### 8.5. Incorporation and Delivery of Essential Oils

The labile and volatile compounds in essential oils can easily evaporate or decompose and are poorly soluble in water, affecting their antioxidant and antibacterial properties. Incorporating essential oils into nanoparticles, such as natural and biodegradable biopolymeric particles, successfully protects the essential oil components from degradation under adverse environmental conditions, extends shelf life, and contributes to controlled release [316,317].

Hadid et al. [316] successfully synthesized chitosan-based nanoparticles loaded with extracted clove bud essential oil, which exhibited better antioxidant and antimicrobial activity against *Listeria monocytogenes* and *S. aureus* compared to free essential oil. Hasheminejad et al. [180] also successfully incorporated clove essential oil into chitosan nanoparticles, which increased antifungal activity against *A. niger*. The in vitro release studies showed a controlled release of the essential oil over 56 days. By successfully encapsulating basil (*Ocimum basilicum* L.) essential oil in chitosan nanoparticles, Cai et al. [181] demonstrated enhanced antibacterial activity and strong antibiofilm activity against *E. coli* and *S. aureus*. Similarly, mandarin (*Citrus reticulata* L.) essential oil was successfully loaded into chitosan-based nanoparticles [318]. The nanoformulation successfully damaged the cell membrane of *E. coli* and *S. aureus* and inhibited biofilm formation and destroyed mature biofilms.

By incorporating essential oil from nettle leaves into chitosan nanoparticles, Bagheri et al. [319] achieved improved antioxidant activity compared to free essential oil and a more effective inhibitory effect on the growth of *E. coli* and *S. aureus*. Soltanzadeh et al. [184] incorporated the essential oil of lemongrass (*Cymbopogon commutatus*) into chitosan nanoparticles and demonstrated a time- and pH-dependent release of the essential oil in an in vitro release study. The chitosan nanoparticles also successfully retained the essential oils and preserved their bioactivity. Compared to free nanoparticles, nanoparticles with incorporated essential oil exhibited stronger antibacterial and antifungal activities. Furthermore, the essential oil of peppermint (*Mentha piperita*) and green tea (*Camellia sinensis*) [320] were also successfully loaded into chitosan nanoparticles, which exhibited enhanced thermal stability, antioxidant and antimicrobial activity. Additionally, cumin (*Cuminum cyminum*) seed essential oil was successfully encapsulated in chitosan nanoparticles [321]. Therefore, chitosan-based nanoparticles have emerged as a novel approach for the production of new pharmaceutical products with antimicrobial activity.

Furthermore, Yoncheva et al. [322] successfully encapsulated oregano essential oil in chitosan-alginate nanoparticles. The thermal stability of the oil, as well as antibacterial and antimetabolic activity compared to pure oil, was improved. In addition, an in vitro cytotoxicity test on human keratinocytes and an in vivo skin irritation test in an animal model demonstrated the safety profile of the oil-loaded nanoparticles. Merino et al. [220] incorporated *Thymbra capitata* essential oil into zein nanoparticles prepared by the self-assembly method and achieved an encapsulation efficiency of 77.8%. Compared with the essential oil in suspension, the essential oil-loaded nanoparticles exhibited enhanced antimicrobial activity. In addition, zein nanoparticles enable the controlled release of essential oils, making them beneficial nanomaterials for pharmaceutical and cosmetic purposes. Cai et al. [323] successfully incorporated tea tree essential oil into gliadin-based nanoparticles stabilized with gum Arabic.

### 8.6. Incorporation and Delivery of Different Therapeutic Substances: A Summary

Many studies have already been conducted testing both protein and polysaccharide nanoparticles of different origins as promising biodegradable and biocompatible delivery systems for drugs and other therapeutic agents. The aim of the studies was the improvement of bioavailability and therapeutic effect, as well as preventing possible adverse side effects of the loaded cargo.

Table 8 shows examples of drugs and other therapeutic agents incorporated into biopolymer nanoparticles, including their potential therapeutic applications.

## 9. Regulatory Aspects of Biopolymer-Based Nanoparticles

Biopolymers play a crucial role in major industrial sectors. Their sales are increasing, however, their demand for biomedical applications will grow rapidly in the coming years.

Various nanoparticles represent successful materials in numerous health applications, but there are still concerns about their safe use and long-term effects on human health. Therefore, risk management and regulatory issues remain [327]. Although biopolymeric nanoparticles are considered safe, biocompatible, biodegradable, and nontoxic, clinical studies on their safe use in drug delivery are urgently needed. Considering the many successful studies on biopolymeric nanoparticles with incorporated therapeutic agents, many companies are already developing suitable biopolymer-based nanoparticle formulations, some of which are already close to pre-clinical and clinical trials or are already in Phase 1. An example of a commercial formulation that is in Phase 1 is DE-310, which consists of a topoisomerase I inhibitor (exatecan mesylate) and a biodegradable carboxymethyl-dextran polyalcohol polymer covalently linked via a Gly-Gly-Phe-Gly peptidyl linker. In a Phase 1 clinical trial, it achieved complete remission in one of 27 patients with metastatic adenocarcinoma studied and partial remission in one patient with metastatic pancreatic cancer. In addition, it led to stabilization of disease progression in 14 patients. Delimotecan (MEN 4901/T-0128) is also a Phase 1 dextran-based nanoformulation with incorporated camptothecin (T-2513) for the treatment of solid tumors. The alginate and calcium gluconate-based formulation IK-5001 is also in Phase 1 for the prevention of ventricular remodeling and congestive heart failure. Another formulation is already in Phase 2, chitosan-based Milican, with incorporated holmium-166 for the treatment of small hepatocellular carcinoma [83].

On the other hand, some formulations containing biopolymeric nanoparticles are already successfully used and approved by the Food and Drug Administration (FDA) for various applications, including cancer treatment [52]. The first nanoformulation containing biopolymeric nanoparticles for the treatment of advanced metastatic pancreatic, breast, and non-small lung cancer is Abraxane, which the FDA has approved. Abraxane is a commercial formulation of the hydrophobic anticancer drug paclitaxel loaded into albumin-based nanoparticles. The nanoformulation is intended for intravenous administration and contains nanoparticles of 135 nm diameter [133]. Yuan et al. [328] investigated the efficacy and mechanism of two commercially available formulations to eliminate cancer stem cells in vitro and in vivo. In their study, they found that Abraxane, which contains paclitaxel-loaded albumin nanoparticles, improved the efficacy of paclitaxel in eliminating breast cancer stem cells over Taxol, a micelle formulation of paclitaxel [133].

However, the regulatory requirements for biopolymeric nanoparticles need greater attention and further research to ensure that commercial formulations can successfully overcome the challenges associated with stability and, more importantly, safety.

## 10. Conclusions

Research in the field of various nanoparticles as delivery systems for drugs and other therapeutic agents for healthcare applications is growing rapidly. However, synthetic nanoparticles can be toxic and have other unfavorable properties. Therefore, nanoparticles derived from natural materials such as biopolymers of animal, plant, algal, fungal, and bacterial origin are receiving increasing attention. Namely, they offer many advantages that are important factors for their potential use as successful and safe nanocarriers for drugs in the human body. Precisely because of their biocompatibility, biodegradability, nontoxicity, antioxidant properties (natural polysaccharides), and stability, research on the potential use of protein- and polysaccharide-based nanoparticles as favorable nanocarriers for therapeutic agents as new forms of treatment has significantly increased in the last decade. Furthermore, biopolymeric nanoparticles enable efficient encapsulation of many therapeutic agents, ensuring successful protection from degradation in the gastrointestinal tract, preventing potential toxic side effects of the loaded therapeutic agents, and ensuring controlled release at the target site, making them particularly promising for oral administration.

This review aims to generally outline the current state of research in the field of promising delivery of biopolymeric nanoparticles loaded with various therapeutic agents, both drugs and antibiotics, as well as natural extracts and bioactive compounds for biomedical applications. Biopolymeric nanoparticles successfully increase the bioavailability and improve the therapeutic effect not only of drugs, antibiotics, and other antimicrobial agents but also of extracts and essential oils, especially naturally isolated bioactive compounds. As far as natural compounds and their incorporation into biopolymeric nanoparticles are concerned, most studies have been conducted on curcumin and other bioactive compounds, which have thus shown promise as nanocarriers for various biomedical purposes. However, different encapsulated extracts and essential oils show significantly enhanced antioxidant and antimicrobial activity. Therefore, nanoparticles prepared from natural biopolymers with incorporated natural extracts and bioactive compounds are a promising alternative to various conventional therapies and therapeutic agents that cause toxic side effects and cytotoxicity to healthy cells. Moreover, they may not reach the target sites at all, leading to drug resistance in patients.

Based on the reviewed literature, studies on the use of polysaccharide nanoparticles for healthcare applications predominate. Chitosan is among the most studied biopolymers for the preparation of nanoparticles for the successful delivery of various therapeutic agents, as demonstrated by numerous in vitro studies. It is one of the most readily available and economical biopolymers and, therefore, the most suitable for commercial application. Nevertheless, further studies are urgently needed because chitosan nanoparticles, despite their excellent potential, are less recommended for therapeutic purposes due to the possible transmission of diseases from animals to humans by animal-based nanoparticles. It should be emphasized that preclinical studies are crucial for potential commercial use. Therefore, it is recommended to focus more on plant and microbial polysaccharide-based nanoparticles. Nowadays, polysaccharides can be largely obtained from algae, which are considered to be of less concern for healthcare use. Although there is a great tendency to cultivate algae for other applications such as food applications, fuels, etc., it is reasonable to direct the cultivation towards the extraction of polysaccharides for the synthesis of algae-based nanoparticles. However, further studies are critically needed to ensure the production of the most therapeutically successful, safe, sustainable, and biodegradable biopolymeric nanoparticles for healthcare applications.

## Figures and Tables

**Table 1:** Examples of proteins used for the production of protein-based nanoparticles.

Origin of Protein	Protein Source	Protein	Ref.
Animals	Ovalbumin and bovine serum albumin	Albumin	[71]
	Milk	Casein	[72]
	Muscles, tendons, skin, bones	Collagen	[73]
	Bones, skin, connective tissue	Gelatin	[74]
	Hair, nails, skin, feathers, wool, hooves, beaks, horns	Keratin	[75]
	Silkworm *Bombyx mori*	Silk fibroin and sericin	[76]
Plants	Wheat	Gliadin	[77]
	Corn kernel	Zein	[78]

**Table 2:** Examples of polysaccharides used for the production of polysaccharide-based nanoparticles.

Origin of Polysaccharide	Polysaccharide Source	Polysaccharide	Ref.
Animal	Shells of arthropods such as crabs, prawns, shrimps, and lobsters	Chitosan	[88]
Plant	*Larix* spp.	Arabinogalactan	[89]
	Agricultural trashes: sugarcane bagasse, rice husks	Cellulose	[37,90]
	Plant sources: wood, cotton, jute, hemp, bamboo, coconut, wheat, rice, grass, agave, flax		[37,90]
	*Cyamopsis tetragonoloba*	Guar gum	[91]
	*Cyamopsis psoraloides*		[92]
	Globe artichoke, topinambur, elecampane, chicory	Inulin	[93]
	Citrus peels, apple pomace	Pectin	[94]
	Potatoes, corn, tubers, fruits, legumes, roots, cereals, rice, yams, banana wheat, sorghum	Starch	[37]
	Plant tissues (trees, cotton)	Cellulose	[95]
Algae	*Nizimuddinia zanardini*	Alginate	[96]
	*Sargassum latifolium*		[97]
	*Cystoseira barbata*		[98]
	*Ascophyllum nodosum*		[99]
	*Laminaria digitata*		[100]
	*Laminaria hyperborea*		[101]
	*Laminaria japonica*		[102]
	*Macrocystis pyrifera*		[103]
	*Sargassum wightii*		[104]
	*Chondrus crispus*	Carrageenan	[105]
	*Sarcothalia crispata*		[106]
	*Gigartina skottsbergii*		[106]
	*Eucheuma denticulatum*		[107]
	*Kappaphycus alvarezii*		[108]
	*Hypnea musciformis*		[109]
Fungi	*Aureobasidium pullulans*	Pullulan	[110]
	*Cytaria harioti*		[111]
	*Cytaria darwinii*		[111]
	*Cryphonectria parasitica*		[111]
	*Termella mesenterica*		[112]
	*Teloschistes falvicans*		[112]
	*Rhodotorula bacarum*		[112]
	*Saccharomyces cerevisiae*	Dextran	[113]
	*Rhizopus oryzae*	Chitosan	[114]
Bacteria	*Lactobacillus plantarum*	Dextran	[115]
	*Weissella confuse*		[116]
	*Weissella cibaria*		[117]
	*Leuconostoc mesenteroides*		[118]
	*Leuconostoc reuteri*		[117]
	*Lactobacillus gasseri*		[119]
	*Sphingomonas elodea*	Gellan gum	[120]
	*Sphingomonas paucimobilis*		[120]
	*Leuconostoc citreum*	Levan	[121]
	*Bacillus subtilis*		[122]
	*Bacillus atrophaeus*		[123]
	*Acinetobacter nectaris*		[124]
	*Xanthomonas campestris*	Xanthan gum	[125]
	*Gluconacetobacter xylinus*	Cellulose	[126]
	*Azotobacter* spp.	Alginate	[127]
	*Pseudomonas* spp.		[127]

**Table 3:** Examples of animal-based biopolymeric nanoparticles for healthcare applications.

Biopolymer	Preparation Method	Particle Size (nm)	Loaded Cargo	Healthcare Application	Ref.
HSA	Self-assembly method	<200.0	Docetaxel	Delivery of anticancer drug	[174]
HSA	High-pressure homogenization	150.4	Prednisolone and curcumin	Drug delivery for the treatment of rheumatoid arthritis	[130]
HSA	Desolvation method	<200.0	Piperine	Drug delivery for the treatment of breast cancer cells (MCF-7)	[175]
	Self-assembly method				
HSA	Coacervation method	85.0–120.0	Imipenem and ciprofloxacin	Delivery of antibiotics	[176]
BSA	Emulsification method	100.0	-	Drug delivery studies	[177]
BSA	Desolvation method	156.0–315.0	Doxorubicin	Delivery of anticancer drug	[178]
BSA	Anti-solvent precipitation method	150.0	Paclitaxel and resveratrol	Delivery of anticancer drug	[134]
BSA	Desolvation method	100.0–200.0	Methylene blue	Delivery of antifungal drugs for the treatment of *Candida* infections	[135]
Collagen	Electrospinning/electrospray method	-	-	Appropriate mimicry of the extracellular matrix for biomedical applications	[142]
Collagen	Film dispersion method	35.2–107.0	Cucurbitacin B	Oral delivery	[179]
Chitosan	Ionic gelation method	106.5	Zinc gluconate	Drug delivery for the treatment of rheumatoid arthritis	[167]
Chitosan	Emulsion-ionic gelation	40.0–100.0	Clove essential oil	Superior inhibitory effect against *Aspergillus niger*	[180]
Chitosan	Ionic gelation method	75.4	Rotigotine	Nose-to-brain drug delivery	[168]
Chitosan	Emulsion-ionic gelation	198.7–373.4	Basil essential oil	Delivery of antibacterial substances	[181]
Chitosan	Ionic gelation method	208.1	Cinnamaldehyde	Delivery of anti-quorum sensing agents	[182]
Chitosan	Ionic gelation method	174.0–898.0	Pomegranate peel extract	Controlled delivery of natural antioxidants and antimicrobials	[183]
Chitosan	Emulsification-ionic gelation method	174.0–293.0	Lemongrass essential oil	Delivery of antibacterial substances	[184]

**Table 4:** Examples of plant-based biopolymeric nanoparticles for healthcare applications.

Biopolymer	Preparation Method	Particle Size (nm)	Loaded Cargo	Healthcare Application	Ref.
Gliadin	Desolvation method	1.0–181.0	Sumatriptan	Brain-targeted drug delivery	[188]
Gliadin	Electrospray method	481.6	γ-Oryzanol	Drug delivery to reduce HT-29 cell proliferation	[191]
Zein	Nanoprecipitation method	120.0–180.0	Resveratrol	Oral drug delivery	[195]
Zein	Liquid–liquid phase separation method	379.4	BMP6-derived peptide	Bone regenerative medicine	[196]
Zein	Phase separation	37.0–112.3	Maytansine	Delivery of anticancer drug	[219]
Zein	Self-assembly method	180.0	*Thymbra capitata* essential oil	Drug delivery	[220]
Pectin	Ionotropic gelation method	700.0–850.0	-	Potential oral drug delivery	[200]
Pectin	Ionic gelation method	482.7	Metformin	Drug delivery for type 2 diabetes mellitus	[221]
Starch	Nanoprecipitation method	500.0	Quercetin	Estimation of release kinetics	[204]
Starch	Nanoprecipitation method	<250.0	Curcumin	Drug delivery for polyphenols	[222]
Starch	Nanoprecipitation method	255.0–396.0	Ginkgo biloba extracts	Oral drug delivery	[223]
Starch	Emulsification method	300.0	5-Fluorouracil	Colon-specific drug delivery	[224]
Starch	Acid hydrolysis and precipitation method	85.1	Paclitaxel	Delivery of anticancer drug	[225]
Starch	Nanoprecipitation method	105.0	Paracetamol	pH-responsive drug delivery system	[205]
Starch	Microemulsification method	282.9	Triphala Churna	Drug delivery of drugs with antimicrobial, antibiofilm, and neuroprotective effects	[226]
Cellulose	Nanoprecipitation method	70.0–365.0	Methylene blue	Sustained and controlled release	[227]
Guar gum	Emulsification method	200.0	Celecoxib	Oral drug delivery	[214]
Inulin	Emulsion solvent evaporation method	224.0–365.0	-	Intracellular stimulation of probiotics	[228]
Inulin	Spray drying method	289.8	Quercetin	Colon-targeted drug delivery	[217]

**Table 5:** Examples of algal-based biopolymeric nanoparticles for healthcare applications.

Biopolymer	Preparation Method	Particle Size (nm)	Loaded Cargo	Healthcare Application	Ref.
Alginate	Ionic gelation method	-	Rifampicin	Drug delivery without systemic toxicity	[244]
Alginate	Emulsification method	279.1	Miltefosine	Antifungal drug delivery	[236]
Alginate	Emulsification method	150.0–270.0	Betamethasone sodium phosphate	Ocular drug delivery	[245]
Alginate	Ionic gelation method	400.0–1000.0	Grape pomace extract	Oral delivery	[246]
Alginate	Electrospray method	228.0	CRISPR plasmids DNA	Gene delivery	[247]
Alginate	Emulsification method	279.1	Bacteriocins from *L. paracasei*	Drug delivery for the treatment of fungal diseases caused by *Candida* spp. and *Cryptococcus* spp.	[236]
κ-Carrageenan	Electrospray method	309.5–663.8	D-limonene	Drug delivery of highly sensitive bioactive agents	[242]
κ-Carrageenan	Emulsification method	211.0	Curcumin	Drug delivery	[248]

**Table 6:** Examples of fungal-based biopolymeric nanoparticles for healthcare applications.

Biopolymer	Preparation Method	Particle Size (nm)	Loaded Cargo	Healthcare Application	Ref.
Pullulan	Nanoemulsification method	98.0	Ursodeoxycholic acid	Drug delivery for neuroprotective effect	[257]
Pullulan	Self-assembly method	86.4–222.3	Mitoxantrone	Delivery of anticancer drug	[253]
Pullulan	Nanoprecipitation method	<200.0	Valsartan	Delivery of cardiovascular agents	[258]
Pullulan	Emulsion solvent evaporation method	200.0	Doxorubicin	Liver-specific drug delivery	[259]
Pullulan	Self-assembly method	185.7	Methotrexate and 10-hydroxycamptothecine	Tumor-targeted drug delivery	[260]
Pullulan	Probe-sonication method	84.1	Erlotinib	Drug delivery for cervical cancer therapy	[261]

**Table 7:** Examples of bacterial-based biopolymeric nanoparticles for healthcare applications.

Biopolymer	Preparation Method	Particle Size (nm)	Loaded Cargo	Healthcare Application	Ref.
Dextran	Spray drying method	-	Rifampicin	Enhanced deep lung delivery	[272]
Dextran	Ionic gelation method	34.0–42.0	-	Ocular drug delivery	[265]
Dextran	Single emulsion evaporation method	99.8–181.3	5, 10, 15, 20-tetraphenyl-21H, 23H-porphyrine	Drug delivery for a tumor cell-targeted photodynamic therapy	[273]
Dextran	Self-assembly method	260.0	Naproxen	Anti-inflammatory drug delivery	[274]
Dextran	Solvent diffusion method	<70.0	Chloroquine diphosphate	Delivery of antimalarial drug	[275]
Dextran	Ionic gelation method	69.3	-	Antibacterial agents	[276]
Xanthan gum	Anti-solvent precipitation	149.0–241.0	Cinnamon bark extract	Controlled delivery of polyphenols	[277]
Levan	Electrohydrodynamic atomization method	82.1	Resveratrol	Drug delivery for wound healing and tissue engineering	[269]
Gellan gum	Nebulization/ionic gelation method	337.1	Resveratrol	Oral drug delivery	[268]
Gellan gum	Ionic gelation method	85.6	Atenol	Delivery of antihypertensive drug	[270]

**Table 8:** Examples of therapeutic agents loaded into biopolymeric nanoparticles for different biomedical purposes.

Incorporated Therapeutic Compound	Biopolymer	Administration Route	Therapeutic Application	Ref.
***Bioactive compounds***				
Sylmarin	Collagen	Intraperitoneal injection	Enhanced neuroprotective effect against acute ischemia/reperfusion injury	[291]
Cucurbitacin B	Collagen	Oral delivery	Enhanced absorption of orally administered Cucurbitacin B	[179]
Curcumin	BSA/dextran	In vitro	Enhancement of cellular antioxidant activity of curcumin in Caco-2 cells	[285]
Curcumin	Zein	Oral administration	Increased oral bioavailability	[324]
Curcumin	Zein	Intravenous injection	Potential drug delivery platform for therapy of glioblastoma	[193]
Beta-carotene	Zein	Oral administration	Enhanced cellular uptake, cytotoxicity, and improved oral biopharmaceutical performance	[298]
Anacardic acid	Zein	In vitro	Enhanced antimicrobial activity	[295]
Quercetin	Zein stabilized with soluble soybean polysaccharide	In vitro	Potential all-natural delivery systems for bioactive molecules	[293]
Morin	Starch	Intravenous injection	Therapeutic efficacy in the treatment of hyperuricemia	[325]
***Conventional drugs***				
Prednisolone and curcumin	HSA	Intravenous injection	Improved in vivo therapeutic effect in rats with rheumatoid arthritis	[130]
Paclitaxel and resveratrol	BSA	Intravenous injection	Improved suppression of tumor growth without systemic toxicity	[66]
Paclitaxel and di-fluorinated curcumin	BSA	Intravenous injection	A promising platform for treating gynecological cancers	[133]
Rotigotine	Chitosan	Nose-to-brain administration	Efficient drug delivery as an alternative to conventional routes	[168]
Zinc gluconate	Chitosan	Intraperitoneal injection	A potential alternative for the treatment of rheumatoid arthritis	[167]
Pirfenidone	Chitosan/alginate	Transdermal administration	Drug delivery for the treatment of idiopathic pulmonary fibrosis	[301]
Rifampicin	Alginate	Oral administration	No systemic toxicity and excellent safety after oral administration of nanoparticles	[244]
Maytansine	Zein	Intravenous injection	Strong anti-A549 tumor cell activity	[219]
Sumatriptan	Gliadin	Nasal administration	Effective crossing of the blood-brain barrier	[188]
Metformin	Pectin	In vitro	Oral administration for management of Type 2 Diabetes Mellitus	[221]
Ibuprofen	Starch	Oral administration	Colon-targeted drug delivery	[326]
Mitoxantrone	Pullulan	In vitro	Inhibition of the growth of bladder cancer cells (MB49)	[253]
Ursodeoxycholic Acid	Pullulan	In vitro	Increased neuroprotective effect	[257]
Paclitaxel	Dextran	Intravenous injection	Potential for C6 glioma treatment	[266]
Chloroquine diphosphate	Dextran	Parenteral administration	Treatment of malaria	[275]
***Antibiotics and antimicrobial agents***				
Colistin	HSA	In vitro	High antibacterial activity against the growth and inhibition of biofilm formation of multidrug-resistant Gram-negative bacterial species	[306]
Tobramycin	Alginate/chitosan	In vitro	Efficient delivery of antimicrobial drug	[310]
ε-Polylysine	Alginate/chitosan	In vitro	Delivery of antibacterial agents	[311]
SET-M33 peptide	Dextran	Intrapulmonary administration	Efficient treatment of lung infection	[312]
***Extracts***				
Pomegranate peel extract	Chitosan	In vitro	Enhanced antioxidant and antimicrobial properties	[183]
Grape pomace extract	Chitosan/alginate	Oral delivery	Improved biological activity	[246]
*Artemisia ciniformis* extract	Alginate	In vitro	Inhibition of proliferation of AGS cells	[314]
*Ginkgo biloba* extract	Starch	In vitro	Sustained release in artificial gastric and intestinal juices	[223]
***Essential oils***				
Oregano essential oil	Alginate/chitosan	Dermal administration	Enhanced antimicrobial activity	[322]
Clove essential oil	Chitosan	In vitro	Improved antioxidant and antibacterial activity	[316]
Basil essential oil	Chitosan	In vitro	Enhanced antimicrobial activity and strong antibiofilm activity	[181]
Mandarin essential oil	Chitosan	In vitro	Enhanced inhibition of biofilm formation	[318]
Lemongrass and peppermint essential oil	Chitosan	In vitro	Enhanced thermal stability, antioxidant, and antimicrobial activity	[184]
Green tea essential oil	Chitosan	In vitro	Enhanced thermal stability, antioxidant, and antimicrobial activity	[320]

## Data Availability

Not applicable.
